# Statistical Interdependence between Daily Precipitation and Extreme Daily Temperature in Regions of Mexico and Colombia

**DOI:** 10.3390/e26070558

**Published:** 2024-06-29

**Authors:** Álvaro Zabaleta-Ortega, Teobaldis Mercado-Fernández, Israel Reyes-Ramírez, Fernando Angulo-Brown, Lev Guzmán-Vargas

**Affiliations:** 1 Unidad Profesional Interdisciplinaria en Ingeniería y Tecnologías Avanzadas, Instituto Politécnico Nacional, Ciudad de México 07340, Mexico; azabaletao1900@alumno.ipn.mx (Á.Z.-O.); ireyesr@ipn.mx (I.R.-R.); 2 Facultad de Ciencias Agrícolas, Universidad de Córdoba, Cra. 6 #77-305, Montería 230002, Colombia; tmercado@correo.unicordoba.edu.co; 3 Departamento de Física, Escuela Superior de Física y Matemáticas, Instituto Politécnico Nacional, Ciudad de México 07738, Mexico; angulo@esfm.ipn.mx

**Keywords:** time series, precipitation, temperature, entropy, synchronization

## Abstract

We study the statistical interdependence between daily precipitation and daily extreme temperature for regions of Mexico (14 climatic stations, period 1960–2020) and Colombia (7 climatic stations, period 1973–2020) using linear (cross-correlation and coherence) and nonlinear (global phase synchronization index, mutual information, and cross-sample entropy) synchronization metrics. The information shared between these variables is relevant and exhibits changes when comparing regions with different climatic conditions. We show that precipitation and temperature records from La Mojana are characterized by high persistence, while data from Mexico City exhibit lower persistence (less memory). We find that the information exchange and the level of coupling between the precipitation and temperature are higher for the case of the La Mojana region (Colombia) compared to Mexico City (Mexico), revealing that regions where seasonal changes are almost null and with low temperature gradients (less local variability) tend to display higher synchrony compared to regions where seasonal changes are very pronounced. The interdependence characterization between precipitation and temperature represents a robust option to characterize and analyze the collective dynamics of the system, applicable in climate change studies, as well as in changes not easily identifiable in future scenarios.

## 1. Introduction

Statistical interdependency can quantify interactions between systems’ elements when they evolve synchronously [1,2,3]. It focuses on assertively quantifying the coupling responsible for collective behavior. One of the most fruitful approaches to understand this phenomenon is Kuramoto’s pioneering study in the 1970s on the phase synchronization analysis of coupled oscillators [4]. A number of studies have applied these notions to synchronization analysis between irregular signals, identifying different coupling levels in several fields, including physical and biological systems [5,6,7]. However, despite its usefulness in studying different systems [8], the complex nature of systems has given rise to mathematical complications in this task [9].

From a practical point of view, the evaluation of the coupling level between complex signals requires the incorporation of different approaches. A group of methods which have proved to be very useful in measuring coupling between irregular signals are those derived from information theory or that are entropy based, whose principal applications have focused on physiological signal analysis [10,11,12,13,14,15,16,17,18,19,20,21,22,23,24,25,26,27] but are also applied in other fields such as finance [28,29,30,31], Earth sciences [32,33,34], and engineering [35], among others.

On the other hand, climate is a complex system whose behavior requires an integrated approach to describe its dynamics [36,37] and especially the characterization of coupling levels between representative variables. In past years, a great variety of coupling measures have been applied in the context of the climate study. For instance, Duane [38] studied meteorological teleconnections, using synchronized chaos, and reported the tendency of two hemispheric subsystems that simultaneously occupy the same regime. Berg et al. [39] analyzed seasonal characteristics of the relationship between daily precipitation intensity and surface temperature in Europe, distinguishing separate precipitation types and stating the dependence between temperature and precipitation. Donges et al. [40] compared measures to analyze climatic teleconnections using a complex network approach. Feliks et al. [41] studied the synchronization between the North Atlantic Oscillation and Oscillatory Climate Modes in the Eastern Mediterranean, identifying a significant synchronization. On the other hand, Gennaretti et al. [42] used the correlation coefficient to evaluate the interdependence of average temperature and precipitation for Canadian Arctic coastal zones, highlighting the importance of including interdependence analysis on climate change scenarios, and Jajcay et al. [43] analyzed the causality and synchronization of the El Niño Southern Oscillation, ENSO, and stated that the understanding of founded discrepancies may be the key to improving the ENSO prediction.

Two of the most important and representative climatic variables are precipitation and temperature because they play a key role in the hydrological behavior of a territory with an impact on events such as floods and droughts, among others [44,45,46]. These variables (as physical phenomena) exchange nontrivial information in their (joint) evolution and are indispensable in the climate characterization. Quantifying the coupling level between climatic variables such as precipitation and temperature represents valuable information to robustly characterize their collective behavior, which is relevant in studies of climate change scenarios. However, as mentioned above, the description and characterization of climate variables have mainly focused on analyzing teleconnections and seasonal relationships. Nonetheless, there is a gap in the interdependence study to quantify shared information between climate variables such as precipitation and daily extreme temperature using robust techniques of synchronization measures, which is covered in this paper. In this work, the interdependence between precipitation and extreme daily temperature (maximum and minimum) is studied by measuring their synchronization level. We start with a statistical description of the time series by exploratory data analysis. The initial approach to the synchronization is studied using the cross-correlation and coherence functions, whereas the deeper analysis is carried out using the mutual information, the global phase synchronization index and the cross-sample entropy.

The remainder of this paper is outlined as follows: Section 2 contains the material and methods, which describe the study area, data, and data treatment for applying the techniques of synchronization measures. Section 3 presents the results and discussions of the obtained values from the applied techniques and their dissertation. Finally, Section 4 includes the conclusions.

## 2. Materials and Methods

### 2.1. Study Area and Data

We studied climatic data from two regions. The first one is the metropolitan area of Mexico City (Mexico), one of the most populated cities in the world, where urban expansion has introduced modifications in the atmospheric energy exchange [47]. Daily records of precipitation, and maximum and minimum temperatures of 14 climatic stations from 1960 to 2020 were studied, i.e., 42 time series each with about 20,000 records obtained from Servicio Meteorológico Nacional (SMN) of the Comisión Nacional del Agua (CONAGUA, https://smn.conagua.gob.mx/es/climatologia/informacion-climatologica/informacion-estadistica-climatologica, last accessed date: 8 May 2024). The second region refers to La Mojana (Colombia), which serves as a hydraulic damping system for the Cauca, San Jorge, and Brazo Loba (a bifurcation of Magdalena River) rivers that convert it in a great interest area due to its natural diversity, hydrological and hydraulic functions, and agricultural importance with particular climate characteristics and social dynamic [48,49]. For this region, the precipitation and maximum temperature daily records of seven (07) climatic stations between 1973 and 2020 were studied, i.e., 14 time series of about 13,500 records each, obtained upon request to the Instituto de Hidrología, Meteorología y Estudios Ambientales (IDEAM, http://dhime.ideam.gov.co/atencionciudadano/, last accessed date: 8 May 2024). In total, 56 time series were studied. The records period for each region was taken according to the data availability. Because of the information’s lack of minimum temperature, it was not possible to study this variable for La Mojana. The general relevant information on climatic stations is shown in Table 1. To visualize the study area, see detailed online information on the geographical location of the stations at this link: https://colab.research.google.com/drive/1ZWVi9hpvi_Q3ZeR4BOhgTat3sR7l_kbN?usp=sharing, last accessed date: 8 May 2024.

### 2.2. Exploratory and Fractal Data Analysis

This aspect was addressed through descriptive statistics, involving position and central tendency measures and dispersion measures, among other statistics measures, following [50,51,52,53]. The missing data were input using reanalysis data obtained from ERA database (https://cds.climate.copernicus.eu/cdsapp#!/dataset/reanalysis-era5-single-levels?tab=form, last accessed date: 8 May 2024). Also, through visual inspection, we identified outliers, and if any existed, we compared them with nearby stations searching for similar records at the occurrence date. If such an event was an extreme one, we validated it, and we replaced it with the ERA register otherwise. In addition, to characterize the temporal organization of the individual (univariate) series, persistence and fractality were analyzed using rescaled range analysis [54] and Higuchi’s fractal dimension [55]. Details of the procedures for calculating the Hurst exponent (*H*) and the Higuchi fractal dimension (*D*) can be found in [56] and [55,57], respectively. Values of 0.5<H≤1.0 indicate persistence (long-term memory), while 0.0≤H<0.5 indicates anti-persistence, and if H=0.5, the fluctuations are neither persistent nor anti-persistent. Similarly, signals with D<1.5 exhibit long-range correlations, while D>1.5 indicates anti-correlations. There is a direct relationship between *H* and *D* that is applicable to self-affine series given by H=2−D, where 1<D<2 [58].

### 2.3. Synchronization Measures

Let *P*, $T_{max}$, and $T_{min}$ be the precipitation, and maximum and minimum temperatures, respectively. We compute the following measures.

#### 2.3.1. Cross-Correlation Function

The cross-correlation function $c_{P,T}\left(\tau \right)$ between *P* and *T* ($T_{max}$ or $T_{min}$ as appropriate) gives a linear synchronization measure between *P* and *T* at a lag τ, expressed as [59,60]:

(1) $$c_{P,T}\left(\tau \right)=\frac{1}{N-\tau }\sum_{i=1}^{N-\tau }\frac{\left(P_{i}-\bar{P}\right)\left(T_{i+\tau }-\bar{T}\right)}{s_{P}s_{T}},$$

where *N* is the time series size, and $\bar{P}$ and $\bar{T}$ represent the mean values. $s_{P}$ and $s_{T}$ denote the standard deviation of *P* and *T*, respectively.

#### 2.3.2. Coherence Function

The coherence function $\Gamma_{P,T}\left(f\right)$ gives a linear synchronization measure in the frequency domain, involving the Fourier transform of the cross-correlation function of *P* and *T*, modulated with its self-spectral [61,62], that is:

(2) $$\Gamma_{P,T}\left(f\right)=\frac{G_{P,T}\left(f\right)}{\sqrt{G_{P,P}\left(f\right)G_{T,T}\left(f\right)}},$$

where $G_{P,T}\left(f\right)=\int_{-\infty }^{\infty }c_{P,T}\left(\tau \right)e^{j2\pi f\tau }d\tau$ is the crossed spectral of *P* and *T*, and $c_{P,T}\left(\tau \right)$ is the mathematical expectation cross-correlation function. $G_{P,P}\left(f\right)$ and $G_{T,T}\left(f\right)$ are the self-spectrals of the mathematical expectation of the autocorrelation function of *P* and *T*, respectively.

#### 2.3.3. Mutual Information

Mutual information MI(P,T) is an entropy-based measure that quantifies the information amount shared between the random variables *P* and *T* with marginal distributions p(P), p(T) and joint distribution p(P,T) computed as [63,64,65]:

(3) $$MI\left(P,T\right)=\sum_{x\in P}\sum_{y\in T}p\left(x,y\right)log\frac{p(x,y)}{p(x)p(y)},$$

The MI(P,T) also gives a stable measure of the information flow of the variables in terms of its synchronization.

#### 2.3.4. Global-Phase Synchronization Index Using Hilbert Transform

This measure is based on analyzing the instantaneous phases $\Delta \phi_{P,T}\left(t\right)$ of the signals *P* and *T*, whose remarkable characteristics are the signal phase analysis, irrespective of their frequency and nonparametric condition, and are defined as [5,15,24,62]:

(4) $$\begin{array}{c} \gamma_{P,T}\left(t\right)=\sqrt{{\langle \cos\left(\Delta \phi_{P,T}\left(t\right)\right)\rangle }^{2}+{\langle \sin\left(\Delta \phi_{P,T}\left(t\right)\right)\rangle }^{2}},\\ \Delta \phi_{P,T}\left(t\right)=\arctan\left[\frac{{\tilde{S}}_{P}\left(t\right)S_{T}\left(t\right)-{\tilde{S}}_{T}\left(t\right)S_{P}\left(t\right)}{S_{P}\left(t\right)S_{T}\left(t\right)+{\tilde{S}}_{P}\left(t\right){\tilde{S}}_{T}\left(t\right)}\right],\\ {\tilde{S}}_{\cdot }\left(t\right)=H\left(P\left(t\right)\right)=PV\frac{1}{\pi }\int_{-\infty }^{\infty }\frac{S_{\cdot }\left(t\right)}{t-\tau }d\tau , \end{array}$$

where ${\tilde{S}}_{\cdot }\left(t\right)$ is the Hilbert transform of the signal (P(t) or T(t) as appropriate), and PV is Cauchy’s principal value.

#### 2.3.5. Cross-Sample Entropy

Cross−sample entropy, here denoted as CSE, is an entropy-based asynchrony measurement that compares the similarity between two time series. CSE depends on three parameters: *m* is the model vector’s length, *r* is the distance tolerance, and *N* is the time series size. To compute CSE, we proceed as follows [13,14,17]: given the time series (signals) u(t)=P(1),P(2),⋯,P(N) and v(t)=T(1),T(2),⋯,T(N) (just *P*, $T_{max}$ or $T_{min}$ as appropriate), we compute $B^{m}\left(r\right)\left(P||T\right)=\frac{1}{N-m}\sum_{i=1}^{N-m}B_{i}^{m}\left(r\right)\left(P||T\right)$, where $B_{i}^{m}\left(r\right)\left(P||T\right)=\frac{1}{N-m-1}\sum_{i,j=1;i\neq j}^{N-m}\Theta (r-||u_{i}^{m}-v_{j}^{m}\left||\right)$, Θ(·) is the Heaviside step function, $\left|\right|u_{i}^{m}-v_{j}^{m}\left|\right|$ is the Euclidean distance between $u_{i}^{m}=P\left(i\right),P\left(i+1\right),\cdots ,P\left(i+m-1\right)$ and $v_{j}^{m}=T\left(j\right),T\left(j+1\right),\cdots ,T\left(j+m-1\right);\;1\leq i,j\leq N-m-1$. Similarly, we calculate $A^{m}\left(r\right)\left(P||T\right)=\frac{1}{N-m}\sum_{i=1}^{N-m}A_{i}^{m}\left(r\right)\left(P||T\right)$, where $A_{i}^{m}\left(r\right)\left(P||T\right)=\frac{1}{N-m-1}\sum_{i,j=1;i\neq j}^{N-m}\Theta (r-||u_{i}^{m+1}-v_{j}^{m+1}\left||\right)$. Finally, the CSE is defined as:

(5) $$CSE\left(m,r,N\right)=-\ln\left(\frac{A^{m}\left(r\right)\left(P||T\right)}{B^{m}\left(r\right)\left(P||T\right)}\right).$$

CSE is zero when the time series are perfectly synchronized, whereas higher values of CSE indicate asynchrony.

### 2.4. Statistical Significance Test for the Synchronization Metrics

To investigate differences in the metrics obtained between the two regions, using the Scipy stats module (https://docs.scipy.org/doc/scipy/reference/stats.html, last accessed date: 8 May 2024), we computed the *t*-Student test, which is a statistic test that, with a defined significance level (or its equivalent confidence level), compares if two independent samples are similar regarding their mean values [66], and the Mann–Whitney test [67], a nonparametric statistic test that compares if two independent variables are dissimilar.

The set of coupling measures described above allows us to quantify the synchronization degree between precipitation and temperature covering both linear (cross-correlation and coherency function) and nonlinear (mutual information, phase synchronization, and cross-sample entropy) information aspects by studying them in the time (cross-correlation function and entropy-based measures), frequency (coherence function), and phase (phase synchronization index) domains. These metrics provide us with valuable information on the joint evolution to characterize and analyze the relationship between these climatic variables further than conventional statistical analysis. All data processing and metrics computations were carried out in Python (https://www.python.org/, last accessed date: 8 May 2024) language, using libraries such as Numpy (https://numpy.org/, last accessed date: 8 May 2024), Scipy (https://scipy.org/, last accessed date: 8 May 2024), EntropyHub (https://www.entropyhub.xyz/, last accessed date: 8 May 2024) and Matplotlib (https://matplotlib.org/, last accessed date: 8 May 2024) for graphical visualization. The results are described below.

## 3. Results and Discussion

### 3.1. Exploratory Data Analysis

**Figure 1:**
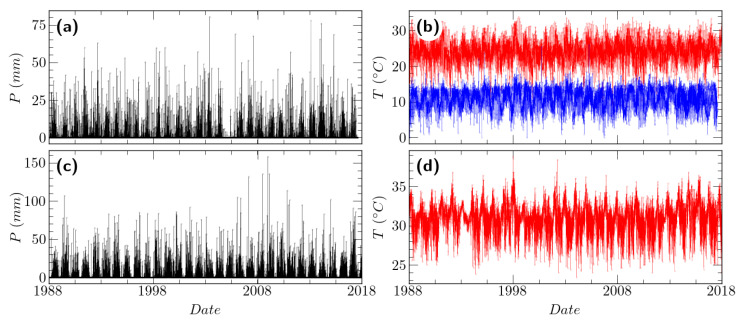
Representative time series of (**a**) precipitation *P* and (**b**) maximum and minimum temperatures *T* ($T_{max}$, red line; $T_{min}$, blue line, respectively) for Mexico City (station MCS13). (**c**) Precipitation *P* and (**d**) maximum temperature *T* ($T_{max}$) for La Mojana (station LMS4).

**Figure 2:**
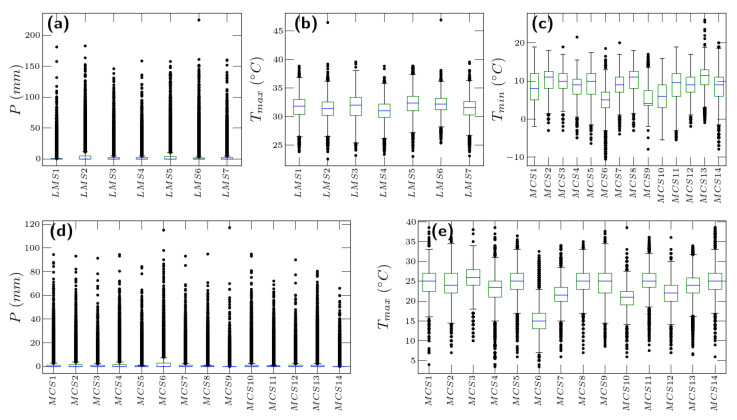
Boxplot of raw data. (**a**,**b**) correspond to the precipitation and maximum temperature, respectively, for La Mojana. (**c**–**e**) correspond to the minimum temperature, precipitation and maximum temperature for Mexico City, respectively. In general, the temperature exhibits more dispersion in Mexico City than in La Mojana, whereas for precipitation, it shows a similar behavior. In all variables, higher event values are observed for La Mojana, which shows important differences in the fluctuations between the two climatic regions.

Figure 1 illustrates representative time series under analysis for both regions. For the analyzed period in Mexico City (1960–2020), the maximum temperature recorded values between 3.5 °C and 38.5 °C, with a mean value of 23.3 °C, the minimum temperature registered values ranging from −10.5 °C to 26.0 °C with a mean value of 8.3 °C, while the precipitation presented the maximum value of 117 mm in 24 h. The maximum temperature exhibits dispersion below the first quartile and above the third quartile, showing higher variability in the extremes. On the other hand, the minimum temperature shows less variability in the extreme values. In La Mojana, for the analyzed period (1973–2020), the maximum temperature ranges from 22.6 °C to 46.9 °C, whose mean value oscillates around 31.6 °C. The highest precipitation event reported has a magnitude of 301.3 mm. In general, the maximum temperature in the Mojana is less dispersed than in Mexico City, with a concentration between the first and third quartiles. Figure 2 shows the boxplot of the analyzed climatic variables.

The data structure was studied through its persistence and fractality. The persistence was analyzed using Hurst exponent *H* obtained with the rescaled range method, which indicates the presence of long-term correlations among the records. For all variables under study and both regions, Mexico City and La Mojana, Hurst values fall within the interval 0.5<H≤1.0 (see Table 2). For precipitation, the magnitude of certain rainfall events has a long-term relationship, and the same applies for the temperature as well. For Mexico City, it is observed that $H_{T_{min}}>H_{T_{max}}>H_{P}$ and for La Mojana, $H_{T_{max}}>H_{P}$. These results indicate that these climatic variables have different levels of long-term correlations, i.e., “process memory”. Moreover, these results agree with other studies in terms of the persistence values for the scaling indexes in different climate analysis [56,68,69,70,71,72,73,74,75].

On the other hand, Table 3 shows the results of the Higuchi fractal dimension (*D*). We find that, in general, $D_{P}>D_{T_{max}}>D_{T_{min}}$, preserving the same hierarchy in the irregularity of the structure in the variables from both regions. When the fractal dimension associated with precipitation is compared between regions, we observe that, in most cases, the one corresponding to Mexico City is larger than the one corresponding to La Mojana, confirming that there is a greater irregularity in the former. Thus, precipitation tends to be a very irregular phenomenon and therefore difficult to predict, while the relative regularity of temperature makes it somewhat more predictable. In addition, the results shown in Table 3 are consistent with the long-term self-correlations presented in Table 2 for the Hurst exponent, and the values satisfy the known H=2−D relationship.

**Figure 3:**
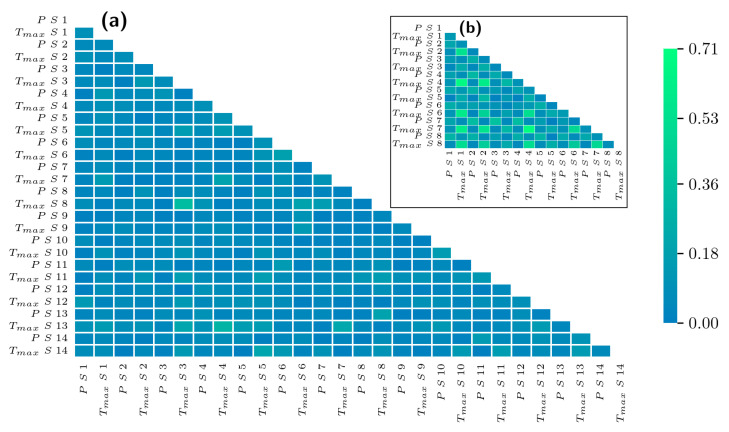
Global Pearson correlation coefficient matrix between precipitation and maximum temperature of empirical data in (**a**) Mexico City and (**b**) La Mojana.

As a general approach of linear correspondence, the global Pearson correlation (which, roughly speaking, is a linear correspondence relationship between two independent variables) between the precipitation and temperature (maximum $T_{max}$ and minimum $T_{min}$ as appropriate) is computed for all the variables for each region (Mexico City an La Mojana), and the results are illustrated in Figure 3. It can be seen from the correlation matrix in Figure 3 that there is a high global relationship between the variables as the climatic zone correspondence.

In general, according to Figure 3, La Mojana exhibits higher values of Pearson correlations than Mexico City. This effect is possibly due to the higher relative stability of climatic variables in La Mojana, which has a stretched interval of occurrence values compared with those from Mexico City.

### 3.2. Synchronization Measures

To reduce the effects of spurious correlations that can affect the applied techniques and lead to misleading results, we normalized the time series before computing the synchronization measures by extracting its mean and dividing by the standard deviation such that the time series are normalized to have zero mean and unitary variance.

#### 3.2.1. Cross-Correlation Function

After exploring the Pearson correlation comparing all the variables between them for each regions, we evaluated the linear synchronization as time dependence through cross-correlation involving the variables (precipitation and temperature) in the same station. The results of the calculations are shown in Table 4. For Mexico City, the highest values of cross-correlation between *P* and *T* occur at lag τ=0 (with global average $c(P,T_{max})=0.228\pm 0.064$ between precipitation and maximum temperature, and $c(P,T_{min})=0.096\pm 0.031$ between precipitation and minimum temperature), meaning that once a rainfall event occurred, the closest-related temperature event occurred on the same day. In contrast, for La Mojana, the highest values occur at lag τ=1 (with global mean value of $c(P,T_{max})=0.256\pm 0.044$ between precipitation and maximum temperature), i.e., once a precipitation event has occurred, the temperature with which it is most closely related occurred on the last day. This result is reasonable when considering the variability of the magnitude of the events in the different regions, being more stable in La Mojana.

#### 3.2.2. Coherence Function

**Figure 4:**
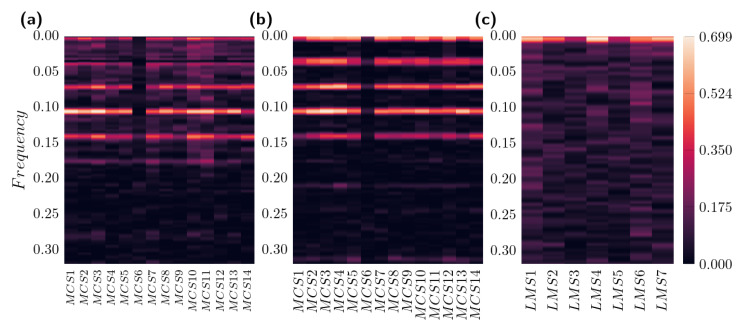
Coherence function heatmap of (**a**) *P* vs. $T_{max}$, (**b**) *P* vs. $T_{min}$ for Mexico City and (**c**) *P* vs. $T_{max}$ for La Mojana.

The coherence function shows several bands of high synchronization at different frequencies for Mexico City (See Figure 4a and Figure 4b corresponding to *P* vs. $T_{max}$ and *P* vs. $T_{min}$, respectively), while La Mojana (Figure 4c for *P* vs. $T_{max}$) has only one frequency band where the synchronization is high. It is reasonable to attribute this behavior to the seasonality effect for Mexico City, i.e., the coherence values are related to its seasonal condition, giving several bands of synchronization in terms of their frequencies. Indeed, because of its lack of seasonality, La Mojana exhibits only one frequency band, suggesting that using specific frequency bands to analyze climate records will lead to a better characterization of climate records. In general, for Mexico City, the global average coherence between *P* and $T_{max}$ is 0.061±0.018 (average ± standard deviation), while between *P* and $T_{min}$, it is slightly higher with a mean value of 0.064±0.026. On the other hand, for La Mojana, the average coherence between *P* and $T_{max}$ has a global mean value of 0.088±0.017. Regardless of the seasonality effect, note that La Mojana exhibits higher global average coherence than Mexico City, meaning more synchronization of the analyzed variables for the former.

#### 3.2.3. Mutual Information

As shown in Table 5, for Mexico City, MI has greater values for *P* and $T_{max}$ than *P* and $T_{min}$. The average values are the following: $MI(P,T_{max})=1.14\pm 0.29$ and $MI(P,T_{min})=1.08\pm 0.26$ (clearly $MI\left(P,T_{max}\right)>MI\left(P,T_{min}\right))$. These results indicate that precipitation shares more information with the maximum temperature than with the minimum one. For La Mojana, the mean value is $MI(P,T_{max})=1.67\pm 0.20$, and, in general, MI exhibits higher values compared to those observed in Mexico City, confirming that both variables share more information for this region.

#### 3.2.4. Phase Synchronization Index of Hilbert Transform

The calculations of the γ-index are shown in Table 6 for both regions. For Mexico City data, similar γ-values are observed when they come from either *P* and $T_{max}$ or *P* and $T_{min}$. We find that La Mojana leads to higher vales compared to Mexico City. In general, according to Table 6, values of $\gamma_{P,T}$ are above 0.72 for Mexico City, whereas for La Mojana, the values are above 0.92.

#### 3.2.5. Cross Sample Entropy CSE

As a synchronic measure, CSE values close to zero mean synchrony, while higher values mean asynchrony. Table 7 shows the obtained results for this measure. In addition, to ensure that the information obtained by this metric comes from the behavior of the time series and not from spurious correlations, we also calculate the CSE for the random (shuffled) version of the time series.

The average $CSE_{E}$ between precipitation and maximum temperature for Mexico City is 1.059±0.276, whereas the average $CSE_{R}$ for randomized time series is 3.491±0.592. There is a similar occurrence between the precipitation and minimum temperature (for Mexico City), where the average values satisfy $CSE_{R}>CSE_{E}$. For La Mojana, the average $CSE_{E}$ between precipitation and maximum temperature is 0.960±0.404, and $CSE_{R}$ has a value of 3.409±0.327. In general, the average $CSE_{E}$ from Mexico City is (about 10%) greater compared with the values obtained from La Mojana, which is in agreement with the results obtained with all previously explored metrics, i.e., a higher synchronization is observed in the latter region.

### 3.3. Statistical Significance Test for the Synchronization Metrics

To distinguish if the synchronization measures are different between the two studied regions, we test the statistical significance of our results for the coupling measures involving precipitation and maximum temperature using *t*-Student and Mann–Whitney tests, stating as a null hypothesis that, with 95% confidence level, the metrics are the same for both regions. The results are presented in Table 8.

Note from Table 8 that, except for cross-correlation, the explored metrics are different between the regions (*p*-value << 0.05) for both *t*-student and Mann–Whitney tests. The cross-correlation is able to measure relationships between two random variables that follow a linear behavior; however, as they do not show to be different between the two regions, this is most likely due to the nonlinearity of the variables studied, which can be considered a manifestation of the higher complexity that characterizes their joint evolution.

## 4. Discussion and Conclusions

We have presented a study, based on linear and nonlinear synchronization measures, to identify the level of coupling between daily precipitation and extreme daily temperature records of climate stations from two regions. We find that the degree of coupling is approximately similar for stations in the same region, while when comparing the two regions analyzed, which have dissimilar climatic characteristics, there is a significant difference (at a confidence level of 95%) in the degree of coupling.

The information presented is consistent and is in agreement with those reported in the literature regarding spatial behavior and complexity [38,39,40,41,42,76,77,78,79]. There is evidence that, in a climatic station, precipitation and daily extreme temperatures share information on its dynamics. At first, the results obtained confirm that precipitation data from the two regions exhibit a persistent behavior and temperature records display even more persistent features. When comparing the records from both regions, it is observed that the persistence is greater in the case of La Mojana, indicating that both precipitation and temperature from Mexico City display higher variability that resembles more erratic variations (less memory).

The global information shared between these variables is evidenced by metrics such as those used in this work; however, due to the nonlinear nature of these relationships, it was found that linear metrics such as cross correlation and coherence do not measure interdependence in a robust way, although they provide some characteristics that allow making analysis decisions such as the selection of bands for the detailed study of the time series in the frequency domain as evidenced by coherence. It was also corroborated that metrics such as mutual information quantify the flow of information between the variables studied, being very significant in this case. Our results show different levels of interdependence between precipitation and temperature, demonstrating that these intensities in the associations between the variables depend strongly on the geographic region and local effects that significantly impact the dynamics of these climatic variables. Particularly, our results have indicated that data from Mexico City exhibit a lower synchrony compared to data from La Mojana. This has been verified in the five metrics used to characterize the interdependence between the signals.

The differences in the level of coupling between the regions studied can be explained in the context of greater variability in the case of Mexico City, where seasonality is very important, while in the Mojana area, this component is almost absent. Further studies that include a number of regions with diverse local conditions are needed to better characterize the zones by the levels of coupling achieved and to determine general patterns that will help to better understand these climatic variables.

Future directions for this type of studies could include the identification of possible precursor patterns of extreme values in the variables that could be linked to a greater coupling between the signals or to a lower synchrony, as well as causality, including the use of strategies of topological data analysis to study the synchronization phenomena as used in [80,81]. In summary, the interdependence between temperature and precipitation is of vital importance for a better understanding of climate dynamics, with implications ranging from the environmental impacts already evidenced by climate change to economic and social consequences.

## Figures and Tables

**Table 1:** General information of climatic stations.

Station	Station Code	Latitude	Longitude	Altitude (msnm)
**Mexico City**				
MCS1	9010	19.4125	−99.2017	2271
MCS2	9014	19.3033	−99.1481	2256
MCS3	9071	19.3339	−99.1322	2250
MCS4	9020	19.2969	−99.1822	2296
MCS5	9029	19.4767	−99.0914	2239
MCS6	9022	19.1344	−99.1731	2990
MCS7	9032	19.1906	−99.0219	2420
MCS8	9036	19.3953	−99.0978	2235
MCS9	9068	19.4292	−99.0528	2240
MCS10	9041	19.1967	−99.1286	2620
MCS11	9043	19.4653	−99.0792	2620
MCS12	9045	19.1789	−99.0028	2240
MCS13	9048	19.4036	−99.1961	2595
MCS14	9051	19.2628	−99.0036	2309
**La Mojana**				
LMS1	25025100	9.28194	−74.84528	18
LMS2	25025150	8.29519	−75.16450	20
LMS3	25015010	8.18078	−75.63228	170
LMS4	25025170	8.74086	−75.49883	125
LMS5	25025240	8.54283	−74.63556	20
LMS6	25025210	8.92075	−74.47425	10
LMS7	25025190	8.39933	−75.58372	90

**Table 2:** Hurst exponent values *H* for *P* ($H_{P}$), $T_{max}$ ($H_{T_{max}}$) and $T_{min}$ ($H_{T_{min}}$).

Mexico City							
**Station**	$H_{P}$	$H_{T_{\max}}$	$H_{T_{\min}}$	**Station**	$H_{P}$	$H_{T_{\max}}$	$H_{T_{\min}}$
MCS1	0.78	0.83	0.85	MCS8	0.78	0.83	0.85
MCS2	0.78	0.83	0.85	MCS9	0.78	0.83	0.85
MCS3	0.78	0.83	0.85	MCS10	0.78	0.83	0.84
MCS4	0.81	0.83	0.84	MCS11	0.78	0.83	0.84
MCS5	0.77	0.83	0.84	MCS12	0.78	0.83	0.85
MCS6	0.78	0.83	0.82	MCS13	0.77	0.77	0.83
MCS7	0.84	0.83	0.83	MCS14	0.78	0.83	0.84
**La Mojana**							
**Station**		$H_{P}$	$H_{T_{\max}}$	**Station**		$H_{P}$	$H_{T_{\max}}$
LMS1		0.83	0.87	LMS5		0.75	0.78
LMS2		0.76	0.82	LMS6		0.77	0.79
LMS3		0.77	0.84	LMS7		0.77	0.81
LMS4		0.74	0.86	----		----	----

**Table 3:** Higuchi fractal dimension *D* values for *P*, $T_{max}$ and $T_{min}$.

Mexico City							
**Station**	$D_{P}$	$D_{T_{\max}}$	$D_{T_{\min}}$	**Station**	$D_{P}$	$D_{T_{\max}}$	$D_{T_{\min}}$
MCS1	1.26	1.21	1.15	MCS8	1.28	1.21	1.15
MCS2	1.26	1.23	1.15	MCS9	1.25	1.21	1.14
MCS3	1.25	1.22	1.14	MCS10	1.27	1.21	1.16
MCS4	1.25	1.22	1.16	MCS11	1.31	1.23	1.15
MCS5	1.30	1.23	1.15	MCS12	1.25	1.23	1.15
MCS6	1.23	1.23	1.27	MCS13	1.28	1.23	1.17
MCS7	1.27	1.23	1.16	MCS14	1.29	1.22	1.14
**La Mojana**							
**Station**		$D_{P}$	$D_{T_{\max}}$	**Station**		$D_{P}$	$D_{T_{\max}}$
LMS1		1.22	1.13	LMS5		1.21	1.15
LMS2		1.19	1.15	LMS6		1.24	1.16
LMS3		1.24	1.17	LMS7		1.23	1.17
LMS4		1.25	1.14	----		----	----

**Table 4:** Cross-correlation values c(P,T) between precipitation *P* and temperature *T* ($T_{max}$ or $T_{min}$ as appropriate) for Mexico City and La Mojana.

Mexico City						La Mojana	
**Station**	$c(P,T_{\max})$	$c(P,T_{\min})$	**Station**	$c(P,T_{\max})$	$c(P,T_{\min})$	**Station**	$c(P,T_{\max})$
MCS1	0.173	0.096	MCS8	0.245	0.063	LMS1	0.307
MCS2	0.254	0.078	MCS9	0.260	0.059	LMS2	0.265
MCS3	0.279	0.102	MCS10	0.187	0.129	LMS3	0.176
MCS4	0.291	0.070	MCS11	0.280	0.080	LMS4	0.266
MCS5	0.259	0.079	MCS12	0.261	0.136	LMS5	0.214
MCS6	0.039	0.169	MCS13	0.215	0.081	LMS6	0.308
MCS7	0.231	0.115	MCS14	0.211	0.087	LMS7	0.255

**Table 5:** Mutual information MI(P,T) for *P* and *T* ($T_{max}$ and $T_{min}$) in Mexico City and La Mojana.

Mexico City						La Mojana	
**Station**	$\mathrm{MI}(P,T_{\max})$	$\mathrm{MI}(P,T_{\min})$	**Station**	$\mathrm{MI}(P,T_{\max})$	$\mathrm{MI}(P,T_{\min})$	**Station**	$\mathrm{MI}(P,T_{\max})$
MCS1	1.65	1.47	MCS8	1.48	1.37	LMS1	1.73
MCS2	1.21	1.13	MCS9	1.40	1.27	LMS2	1.93
MCS3	1.60	1.48	MCS10	1.64	1.47	LMS3	1.45
MCS4	1.20	1.09	MCS11	1.20	1.08	LMS4	1.69
MCS5	1.16	1.09	MCS12	1.18	1.14	LMS5	1.57
MCS6	0.99	0.90	MCS13	2.00	1.83	LMS6	1.44
MCS7	1.36	1.22	MCS14	1.03	0.98	LMS7	1.91

**Table 6:** Phase synchronization index γ(P,T) using Hilbert transform between precipitation *P* and temperature *T* ($T_{max}$ or $T_{min}$) for Mexico City and La Mojana.

Mexico City						La Mojana	
**Station**	$\gamma (P,T_{\max})$	$\gamma (P,T_{\min})$	**Station**	$\gamma (P,T_{\max})$	$\gamma (P,T_{\min})$	**Station**	$\gamma (P,T_{\max})$
MCS1	0.739	0.741	MCS8	0.743	0.733	LMS1	0.927
MCS2	0.742	0.733	MCS9	0.740	0.735	LMS2	0.926
MCS3	0.739	0.736	MCS10	0.747	0.736	LMS3	0.925
MCS4	0.744	0.739	MCS11	0.748	0.742	LMS4	0.929
MCS5	0.739	0.736	MCS12	0.745	0.739	LMS5	0.925
MCS6	0.728	0.739	MCS13	0.743	0.738	LMS6	0.926
MCS7	0.745	0.737	MCS14	0.742	0.732	LMS7	0.926

**Table 7:** Cross-sample entropy CSE ($CSE_{E}$ for experimental and $CSE_{R}$ for randomized time series) for *P* and *T* ($T_{max}$ and $T_{min}$ as appropriate) in Mexico City and La Mojana, setting m=6 and r=0.20.

Mexico City									
**Station**	$\left(P,T_{\max}\right)$		$\left(P,T_{\min}\right)$		**Station**	$\left(P,T_{\max}\right)$		$\left(P,T_{\min}\right)$	
	${\mathrm{CSE}}_{E}$	${\mathrm{CSE}}_{R}$	${\mathrm{CSE}}_{E}$	${\mathrm{CSE}}_{R}$		${\mathrm{CSE}}_{E}$	${\mathrm{CSE}}_{R}$	${\mathrm{CSE}}_{E}$	${\mathrm{CSE}}_{R}$
MCS1	1.385	3.288	0.837	2.010	MCS8	0.894	3.823	0.791	2.093
MCS2	0.657	3.950	0.421	1.901	MCS9	0.791	3.474	0.668	3.051
MCS3	0.797	3.150	0.664	2.802	MCS10	0.824	3.548	1.059	3.298
MCS4	1.744	2.552	0.987	2.792	MCS11	1.062	3.567	1.065	3.164
MCS5	1.089	3.779	0.602	4.562	MCS12	1.048	4.025	1.192	3.535
MCS6	1.170	2.944	1.411	2.315	MCS13	1.169	3.407	1.315	3.703
MCS7	1.058	2.583	1.030	3.075	MCS14	1.136	4.790	0.779	2.849
**La Mojana**									
**Station**				$\left(P,T_{\max}\right)$					
				${\mathrm{CSE}}_{E}$			${\mathrm{CSE}}_{R}$		
LMS1				0.437			3.675		
LMS2				0.966			3.602		
LMS3				1.337			3.751		
LMS4				1.590			3.564		
LMS5				0.435			3.105		
LMS6				0.813			3.394		
LMS7				1.142			2.772		

**Table 8:** List of *p*-values to compare the statistical differences between the synchronization measures from the two regions (Mexico City and La Mojana), for precipitation and maximum temperature at a confidence level of 95%.

Coupling Measure	*p*-Value (*t*-Student)	*p*-Value (Mann–Whitney)
Cross-Correlation	0.3182	0.2872
Coherence	0.0019	0.0056
Global Phase Synchronization Index	4.62E-27	0.0003
Mutual Information	0.0050	0.0100
Cross-Sample Entropy	3.25E-11	0.0003

## Data Availability

Data used in this investigation were obtained from Servicio Meteorológico Nacional of the Comisión Nacional del Agua (CONAGUA, https://smn.conagua.gob.mx/es/climatologia/informacion-climatologica/informacion-estadistica-climatologica, last accessed date: 8 May 2024) and Instituto de Hidrología, Meteorología y Estudios Ambientales (IDEAM, http://dhime.ideam.gov.co/atencionciudadano/, last accessed date: 8 May 2024), were they are public available and can be downloaded under the station code.
